# The Influence of Maternal Aerobic Exercise, Blood DHA and EPA Concentrations on Maternal Lipid Profiles

**DOI:** 10.3390/ijerph19063550

**Published:** 2022-03-16

**Authors:** Cody J. Strom, Samantha M. McDonald, Mary-Margaret Remchak, Kimberly A. Kew, Blake R. Rushing, Joseph A. Houmard, David A. Tulis, Roman Pawlak, George A. Kelley, Lisa Chasan-Taber, Edward Newton, Christy Isler, James DeVente, Madigan Raper, Linda E. May

**Affiliations:** 1 Department of Kinesiology and Sport, University of Southern Indiana, Evansville, IN 47712, USA; 2 School of Kinesiology and Recreation, Illinois State University, Normal, IL 61790, USA; smmcdo4@ilstu.edu; 3 Department of Kinesiology & Health, Rutgers University, New Brunswick, NJ 08901, USA; mer253@scarletmail.rutgers.edu; 4 Department of Biochemistry and Molecular Biology, Brody School of Medicine, East Carolina University, Greenville, NC 27834, USA; kakaplan@fbi.gov; 5 Department of Nutrition, Nutrition Research Institute, University of North Carolina-Chapel Hill, Kannapolis, NC 28081, USA; blake_rushing@unc.edu; 6 Department of Kinesiology, College of Health and Human Performance, East Carolina University, Greenville, NC 27858, USA; houmardj@ecu.edu (J.A.H.); mayl@ecu.edu (L.E.M.); 7 East Carolina Diabetes and Obesity Institute, East Carolina University, Greenville, NC 27858, USA; 8 Department of Physiology, Brody School of Medicine, East Carolina University, Greenville, NC 27834, USA; tulisd@ecu.edu; 9 Department of Nutrition Science, East Carolina University, Greenville, NC 27858, USA; pawlakr@ecu.edu; 10 Department of Epidemiology and Biostatistics, West Virginia University, Morgantown, WV 26505, USA; gkelley@hsc.wvu.edu; 11 Department of Biostatistics & Epidemiology, University of Massachusetts, Amherst, MA 01003, USA; lct@schoolph.umass.edu; 12 College of Obstetrics and Gynecology, Brody School of Medicine, East Carolina University, Greenville, NC 27834, USA; newtoned53@gmail.com (E.N.); islerc@ecu.edu (C.I.); deventeja@ecu.edu (J.D.); 13 Edward Via College of Osteopathic Medicine, Blacksburg, VA 24060, USA; mraper@vt.vcom.edu; 14 School of Dental Medicine, Department of Foundational Sciences and Research, East Carolina University, Greenville, NC 27834, USA

**Keywords:** pregnancy, exercise, DHA, EPA, aerobic, lipids

## Abstract

Exercise and polyunsaturated fatty acid (PUFA) supplementation independently improve lipid profiles. The influence of both exercise and PUFAs on lipids during pregnancy remains unknown. This study evaluated exercise, docosahexaenoic acid (DHA) and eicosapentaenoic acid (EPA) concentrations on lipids during pregnancy. Participants were randomized to aerobic exercise or control groups. From 16 weeks gestation until delivery, groups met 3x/week; exercisers performed moderate-intensity aerobic activity, controls performed low-intensity stretching and breathing. At 16 and 36 weeks’ gestation, maternal blood was analyzed for lipids (total cholesterol (TC), high-density lipoprotein (HDL), low-density lipoprotein (LDL), triglycerides (TG)), DHA and EPA. In intent-to-treat analysis, the aerobic group (*n* = 20), relative to controls (*n* = 10), exhibited a higher HDL change across gestation (*p* = 0.03). In a per protocol analysis, the aerobic group, relative to controls, exhibited 21.2% lower TG at 36 weeks (*p* = 0.04). After controlling for 36-week DHA and EPA, exercise dose predicts 36 weeks’ TG (F (1,36) = 6.977, *p* = 0.012, r^2^ = 0.16). Aerobic exercise normalizes late pregnancy TG. During pregnancy, exercise dose controls the rise in TG, therefore maintaining normal levels. DHA and EPA do not have measurable effects on lipids. Regardless of PUFA levels, exercise at recommended levels maintains appropriate TG levels in pregnant women. Normal TG levels are critical for pregnancy outcomes, and further studies are warranted to investigate this association in broader populations.

## 1. Introduction

Due to the progressive increase in maternal estrogen with pregnancy, maternal fat deposits switch from anabolic to catabolic states to maintain appropriate development of the fetus and placenta [1,2]. This switch to catabolism increases the breakdown of fat stores, which increases total cholesterol (TC) and triglycerides (TG) available for maternal and fetal utilization. Further, it is essential for a normalized maternal lipid profile to ensure healthy fetal development and pregnancy outcomes. An abnormal maternal lipid profile in early pregnancy is defined as TG > 150 mg/dL, or TC > 200 mg/dL, or HDL < 50 mg/dL, or LDL > 130 mg/dL [3]. Although there are no specific ranges from early, mid, to late pregnancy, obstetricians typically ensure LDL is <190 mg/dL [4]. Furthermore, studies have suggested normal ranges fall within the 95th percentiles of distribution for TC, low-density lipoprotein (LDL), and TG, while trying to ensure being in the 5th percentile for high-density lipoprotein (HDL) [5,6,7].

Abnormal maternal lipid levels are associated with incidences of adverse pregnancy outcomes, such as gestational diabetes mellitus, preeclampsia, fetal macrosomia, and placental dysfunction. [3,4,5,6,7,8,9,10,11,12,13,14]. Throughout pregnancy, maternal lipids progressively rise to provide requisite nutrient-energy to the mother and fetoplacental unit. Thus, optimal fetal growth and pregnancy outcomes hinge on a healthy maternal lipid profile during the prenatal period [1,2].

In non-pregnant populations, studies demonstrate that adequate physical exercise improves lipid profiles within clinically healthy ranges via decreases in total cholesterol (TC), low-density lipoproteins (LDL), and triglycerides (TG), with increases in high-density lipoprotein (HDL) levels [15,16,17]. Although the American College of Obstetrics and Gynecology (ACOG) strongly recommends most pregnant women to engage in at least 150 min of moderate intensity exercise per week [18,19], little is known about its effects on maternal lipids throughout pregnancy. McDonald et al. (2021) suggest that doses exceeding the ACOG guidelines of 500 MET∙min∙week^−1^ are required to elicit positive effects on maternal health outcomes [20]. A 12-week intervention study observed moderate-to-vigorous combination (aerobic and resistance) exercise significantly decreases TC, LDL and TG and increases HDL in early 3rd trimester [21]. Another study that implemented a 24-week intervention study observed participants achieving 150 min of moderate intensity aerobic activity per week had higher TG levels at 36 weeks; however, the samples were non-fasted [22]. 

Research also shows that dietary supplementation of polyunsaturated fatty acids (PUFAs), particularly docosahexaenoic acid (DHA) and eicosapentaenoic acid (EPA), during pregnancy controls the progressive rise in maternal TG within normal ranges and additionally increases HDL [23,24]. Moreover, studies previously demonstrated that DHA and EPA, via consuming fish or dietary supplements during pregnancy, reduces adipocyte inflammation, oxidative stress, and free radical accumulation leading to lower TC, LDL, and TG, while increasing HDL [23,25,26,27,28,29]. 

Research suggests that prenatal exercise and dietary supplementation of DHA and EPA independently show positive effects on maternal lipids. However, to our knowledge, no studies have investigated the potential relationship between prenatal exercise and PUFA concentrations on late pregnancy maternal lipid profiles. Therefore, the purpose of this study was to examine the influence of prenatal aerobic exercise and maternal concentrations of DHA and EPA on lipid profiles in late pregnancy. We hypothesized that: (1) exercisers will have improved maternal lipid levels; (2) both prenatal aerobic exercise and higher concentrations of DHA and EPA would be associated with healthy maternal lipid levels; and (3) prenatal aerobic exercise and DHA or EPA concentrations would predict improved maternal lipid levels.

## 2. Materials and Methods

All protocols were approved in accordance with the declaration of Helsinki by the East Carolina University Institutional Review Board as part of a larger, longitudinal randomized controlled trial (ECUIRB#12-002425). 

### 2.1. Study Design

We conducted a secondary analysis using data from a 24+ week, prospective, partially blinded, randomized controlled exercise intervention trial conducted between 2015 and 2018. The primary outcomes of the parent trial were the effects of prenatal exercise on fetal and neonatal cardiovascular function [18,30]. The current study focused on evaluating the effects of prenatal exercise and maternal DHA and EPA concentrations on maternal lipid profiles in late pregnancy. All protocols for the study were approved by the East Carolina University Institutional and Vidant Medical Center Review Boards.

### 2.2. Participant Recruitment and Randomization

Pregnant women were recruited from local obstetric clinics in Eastern North Carolina. Other recruitment methods included flyers posted in local businesses, social media, and word-of-mouth. Women eligible for the study if they were: (1) ≤16 weeks pregnant; (2) pregnant with only 1 fetus; (3) aged between 18 and 40 years; (4) between 18.5 and 39.99 kg/m^2^ for body mass index (BMI); (5) cleared to participate in moderate-intensity exercise by their obstetric provider using a PARmed-x for pregnancy [31]; (6) absent of chronic disease; and (7) not currently using alcohol, tobacco, illicit drugs or taking medications negatively impacting fetal growth. After consent and receipt of their obstetric provider clearance letter, the baseline study visits consisting of a submaximal exercise treadmill test and blood draw were conducted. Then, using computer-generated randomization (GraphPad) presented in sealed, sequentially-numbered envelopes, participants were assigned to the moderate-intensity aerobic exercise or light-intensity stretching and breathing group. 

### 2.3. Maternal Peak Aerobic Capacity

Maternal peak aerobic capacity (VO_2peak_) was measured via a submaximal treadmill exercise test following the modified Balke treadmill (Trackmaster 425, CareFusion, Newton, KS, USA) protocol to 85% of heart rate (HR) maximum, previously validated in pregnant women [32]. Maternal carbon dioxide production and oxygen consumption were measured via indirect calorimetry (ParvoMedics, Salt Lake City, UT, USA). These measures, and rating of perceived exertion (RPE) and HR, were used to ensure VO_2peak_ was achieved. Based on HR, maternal age, fitness level, the published prediction formula for VO_2peak_ in pregnancy were utilized [32]. From the HR and VO_2peak_ treadmill test results, estimated individual target heart rate (THR) ranges associated with moderate intensity exercise (40–59% VO_2peak_) were calculated [32]. 

### 2.4. Exercise Intervention

Participants completed the intervention from 16 weeks’ gestation until delivery and were scheduled on three days each week based on their personal availability. Participants received one-on-one supervised exercise training for all exercise sessions. Participants wore heart rate monitors (Polar FS2C HR monitor) throughout each session to track adherence to the prescribed exercise intensity. Heart rate (via Polar monitor) and blood pressure (via manual auscultation) were measured prior to and following each exercise session. All participants began their sessions with 5 min of light-intensity treadmill walking (speed ~3.0 mph), 50 min of their prescribed protocol, then 2–3 min of cool-down. Given the nature of the study, neither participant nor their exercise trainer were blinded to group allocation. 

#### 2.4.1. Aerobic Exercise Group

For aerobic training (AT), participants performed 50 min of continuous, moderate-intensity aerobic exercise, using aerobic exercise equipment of their choice (e.g., treadmill, elliptical, rowing, cycle ergometer). A two-week transition period was assigned starting participants at 30 min of moderate intensity exercise and progressing 5 min in each exercise session until 50 min was achieved. 

#### 2.4.2. Stretching and Breathing Comparison Group

Participants in the stretching and breathing comparison group (CON) engaged in 50 min of guided stretching and breathing techniques. Prescribed stretches targeted all major muscle groups and breathing exercises focused on controlled inhalation and exhalation during each stretch. The CON participants also wore HR monitors to track their adherence to maintaining the performance of light-intensity (<30% VO_2peak_) stretching and breathing. This type of control group helps to ensure comparison with moderate intensity exercise while maintaining participant retention.

#### 2.4.3. Exercise Dose

Exercise dose, defined as the amount of energy expended during exercise, was calculated following each exercise and stretching and breathing sessions for the AT and CON groups, respectively. Each activity performed was assigned its respective metabolic equivalent (METs; energy expended by the skeletal muscles above resting levels). Assigned METs were previously published in the Compendium of Physical Activities [33]. Following, the assigned METS were multiplied by the duration (minutes) and frequency (number of days) of each activity performed. Exercise dose was expressed as MET∙min∙week^−1^ and averaged over the duration of the intervention.

#### 2.4.4. Exercise Adherence and Compliance

Exercise adherence referred to the proportion of exercise or stretching and breathing sessions attended, tracked electronically via REDCap [34], and was calculated by dividing the number of sessions attended by the total number of possible sessions within a participant’s gestational period. Participants were considered “exercise adherent” if their attendance was ≥80%. Exercise compliance referred to the participant’s compliance with the prescribed dose of exercise in the AT and CON group. The AT group was assigned an exercise dose of at least 500 MET∙min∙week^−1^ whereas the CON group was assigned 375 MET∙min∙week^−1^. Participation in physical activity outside of the exercise or stretching and breathing sessions were measured monthly via the Modified Physical Activity Questionnaire. Participants in the AT group failing to achieve the ≥500 MET∙min∙week^−1^ and those in the stretching and breathing group exceeding 375 MET∙min∙week^−1^ were considered “non-compliant”.

### 2.5. Maternal Blood Sample Collection

Maternal blood samples were drawn via venipuncture and fingerstick techniques at 16 and 36 weeks of gestation to measure maternal DHA, EPA, and lipid profiles. From the venipuncture, about 4 mL of blood was collected. The blood samples were collected in anticoagulant tubes and centrifuged at 1000× *g* to separate plasma and red blood cells (RBCs). All blood draws occurred between 6:00 and 8:00 am, controlling for the effect of circadian rhythms. Additionally, participants were instructed to fast overnight (>9 h) and consume plenty of water prior to the blood draw.

### 2.6. Maternal Lipids

Maternal lipids including TC, LDL, HDL, non-HDL, TC/HDL, and TG were analyzed with a Cholestech LDX Analyzer (Alere Inc., Waltham, MA, USA) using specific Lipid Profile•GLU cassettes (Alere Inc., Waltham, MA, USA). The Lipid Profile•GLU cassettes used 40 μL of whole blood collected via the fingerstick technique in lithium heparin-coated capillary tube and calculated maternal lipid within 6 min. The Cholestech LDX Analyzer demonstrates high validity for estimating maternal lipids (Passing-Bablok regression TC = 0.97, HDL = 0.95, and TG = 0.99) and is verified by the Center for Disease Control and Prevention (CDC) Cholesterol Reference Method Laboratory Network [35]. 

### 2.7. Maternal DHA and EPA

Since we did not collect information regarding maternal supplement use (i.e., fish oils, anti-inflammatory substances), we measured DHA and EPA levels on RBCs. The level of PUFAs on RBCs is a direct measure of what is in their body rather than the confounding plasma levels, which only provides information based on long-term intake.

#### 2.7.1. Solid Phase Extraction (SPE)

A 3:1 Optima grade H_2_O (Fisher Scientific, Hampton NH) to RBC solution was vortexed and homogenized, then underwent three series of freeze/thaw cycles, placed in a 100% isopropanol (IPA), frozen in a dry ice bath, thawed in a 35 °C bath, and vortexed again. Aliquots of 400 µL of thawed hemolyzed RBCs were diluted to a 1 mL solution with 500 µL of methanol (MeOH), 90 µL of H2O, and 10 µL of an internal standard solution of deuterated DHA (DHA-d5) and EPA (EPA-d5). DHA-d5 and EPA-d5 were used as internal standards prepared at 0.5 mg/mL in ethanol. The deuterated DHA and EPA solution was controlled for extraction recovery, injection of the mass spectrometer, and ionization variability. A Biotage Pressure+ manifold (Biotage, Charlotte, NC, USA) with Strata-X reversed-phase SPE columns (Phenomenex, Torrance, CA, USA) and positive pressure (1 to 25 psi) was used to extract the RBC supernatants. After conditioning the columns with 1 mL of MeOH and equilibrating with 1 mL of H_2_O twice each, the RBC supernatants were loaded onto the columns; columns were washed with 1 mL of 10:90 MeOH: H_2_O. The organic fraction of metabolites was eluted with 1 mL of MeOH and 1 mL of 60:20:20 Acetonitrile (ACN): IPA: MeOH twice each. Eluates were then evaporated at 40 °C using a steady flow of nitrogen gas and were then reconstituted in 100 µL of 50:50:0.01 H_2_O/MeOH/formic acid for analysis. A method blank was extracted using the same method to aid in background subtraction and identification of contaminants.

#### 2.7.2. Generation of Calibration Curve Standards 

Stock solutions were prepared in ethanol with DHA and EPA standards, each at a concentration of 25 mg/mL. Working standard solutions for DHA and EPA were prepared by serial dilution of the stock solutions in ACN to create primary standards at 0.01, 0.05, 0.1, 0.5, 1, 2.5, 5.0, and 7.5 mg/mL and were inoculated with the same internal standard as the samples. The stock solutions were processed and extracted using the same method of the hemolyzed RBC solution.

#### 2.7.3. Liquid Chromatography/Mass Spectrometry (LC/MS/MS)

Sample extracts were run on an AB SCIEX 3200 triple quadrupole mass spectrometer in negative ionization mode on a Kinetex 2.6 µm C18 100 Å, LC Column 50 × 3 mm. A gradient was used to separate the compounds using mobile phase A: 95:5 water with 0.1% formic acid:methanol and mobile phase B: methanol. Samples were separated using a linear gradient from 10%B to 90%B over 15 min with a flow rate of 0.300 mL/min. Samples (20 µL injections) were analyzed on the LC/MS/MS with a column temperature of 40°C. The mass spectrometer parameters, including the declustering potentials and collision energies, were optimized for DHA (283.2/229.2) and EPA (257.2/203.3). Analyst 1.6.2 (Applied Biosystems) and Multiquant (Applied Biosystems) were used to collect and analyze data. The external standards calibration curve utilized a linear regression model for response ratios (peak area of analyte/peak area of internal standard) to determine the amount of DHA and EPA in the samples. The signal-to-noise ratio (S/N) was used to determine the limit of detection (LOD; S/N ≥ 3) and the limit of quantitation (LOQ; S/N ≥ 10). Difference values for DHA and EPA were calculated between timepoints by subtracting 16-week from 36-week values.

### 2.8. Maternal Descriptors and Covariates

Maternal age, parity, gravida, pre-pregnancy body mass index (BMI), gestational weight gain (GWG), and gestational age were abstracted from various sources including pre-screening eligibility questionnaires and electronic health records. Gestational weight gain was calculated using the standard expression: GWG (lbs) = ([weight_at delivery_] − [weight_before pregnancy_]). In cases of missing weight at delivery data, the last recorded study weight (36 weeks of gestation) was used. Pre-pregnancy BMI was calculated using self-reported height (m), and weight (kg) via the following established equation [36]: BMI = ((weight (kg)) ÷ ([height (m^2^)]))

### 2.9. Statistical Analysis

Between-group mean differences for maternal descriptive characteristics, 16-, and 36-week measurements of maternal lipids, DHA, and EPA, were assessed using two-tailed student independent *t*-tests or Wilcoxon Rank Sum tests, depending on the conditional distribution of the data. Additionally, changes in maternal lipids DHA, and EPA were calculated via the differences in values measured at 16 weeks and 36 weeks of gestation. Intention-to-treat (ITT) and per protocol analyses were performed. ITT included all participants with complete data. Per protocol included participants that were “exercise adherent” to >80% of exercise sessions and dose, and excluded one control participant that did not follow her group assignment. Spearman correlations were performed to assess associations between maternal exercise dose (MET∙min∙week^−1^), maternal DHA, EPA, and lipids at 36 weeks’ gestation and the change in maternal lipids DHA or EPA values. To determine the effect of maternal exercise, and DHA and EPA concentrations, in late pregnancy on maternal lipid levels, multiple linear regression analyses were performed. The primary outcome variables were maternal levels of TC, LDL, HDL, non-HDL, TC:HDL ratio, and TG at 36 weeks of gestation, and the change in levels from 16 to 36 weeks of gestation. The primary independent variables were maternal exercise dose, and DHA and EPA concentrations. The effects of maternal exercise and DHA or EPA were assessed via interaction terms in the regression models. Maternal characteristics assessed as potential covariates were maternal age, pre-pregnancy BMI, gravid, parity, VO_2peak_, GA, GWG, and 16-week gestation, and differences in lipid measures were considered in models. The analysis for each primary outcome followed the same structured series of model building: Model 1 = unadjusted main effects model, Model 2 = Model 1 + interaction (inclusion of exercise OR PUFA levels), and Model 3 (depending on the influence of the interaction) = Model 1 or 2 + maternal covariates. Each maternal covariate was entered into the model separately to evaluate its influence on the parameter estimates. Baseline values of each outcome variable were adjusted for their respective regression models. Statistical analyses were performed using SPSS, version 25 (IBM, Cary, NC, USA). Two-tailed statistical significance was set a priori at α < 0.05. 

#### Statistical Power Analysis

For between-groups comparisons, a post hoc power analysis revealed we would need 30 (15 per group) for the intention-to-treat analysis and 4 (2 per group) for the per protocol analysis with 80% power and alpha level of 0.05 to detect differences in maternal TG.

## 3. Results

### 3.1. Participant Recruitment and Retention

**Figure 1:**
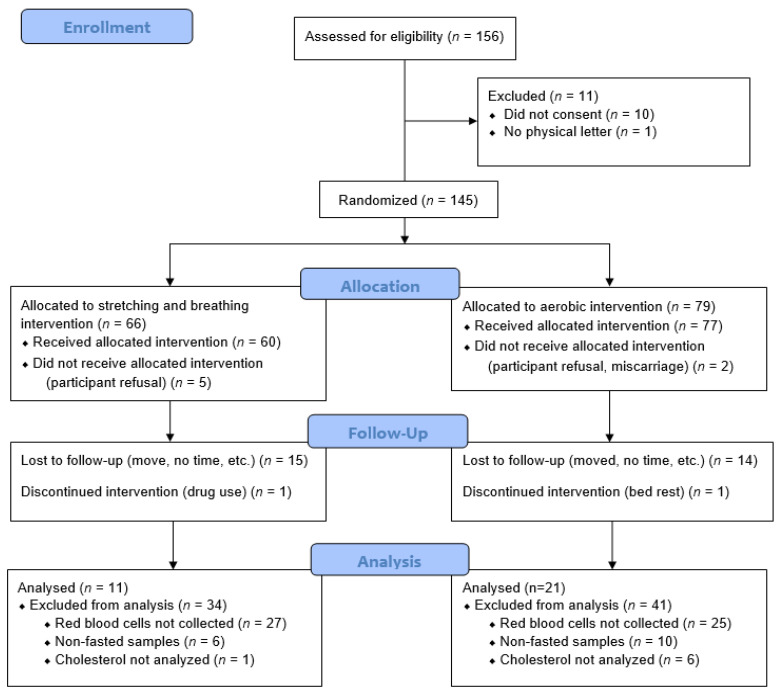
CONSORT diagram.

For this analysis, 156 pregnant women expressed interest and consented (Figure 1). Of these, 145 were randomized to the moderate-intensity aerobic exercise group (*n* = 79) or the control group (*n* = 66). Throughout the study, 38 participants were lost-to-follow-up with participant refusal (*n* = 6), moved, no time or lost interest (*n* = 29), discontinued due to drug use (*n* = 1), discontinued due to bed rest (*n* = 1), or miscarried (*n* = 1). Of the remaining 107 participants, 75 were excluded due to missing data for RBCs and/or lipids (*n* = 59) and non-fasted samples (*n* = 16), yielding a final sample of 32 pregnant women (aerobic = 21, control = 11) eligible for the intent-to-treat analyses. One exerciser was not “exercise adherent” and one control participant did not adhere to her protocol MET level; thus, the per protocol analysis at 36 weeks left 20 aerobic exercisers and 10 control women. 

### 3.2. Study Population

On average, participants were 31 years old, had a pre-pregnancy BMI of 24.8 kg/m^2^ (healthy), and gained 28.9 lbs. All participants delivered full-term (37–41 weeks) infants. The exercise group gained more weight compared to controls (32.7 ± 1.9 vs. 22.6 ± 2.7, *p* = 0.005) during pregnancy (Table 1). The exercise group had a longer GA compared to controls (40.1 ± 0.2 vs. 39.2 ± 0.3, *p* = 0.03) (Table 1). No other between-group differences were observed. 

### 3.3. Maternal DHA, EPA, and Lipid Levels

No between-group differences were observed in maternal concentrations of DHA and EPA at 16 or 36 weeks, or the difference value across pregnancy (Table 2); however, at 36 weeks’ gestation, exercisers had 2.6% higher levels of EPA and 40.9% higher levels of DHA relative to controls. For the ITT protocol, control women exhibited higher maternal TC (211.8 ± 11.0 vs. 177.8 ± 7.9, *p* = 0.01) and TC/HDL (3.3 ± 0.2 vs.2.8 ± 0.1, *p* = 0.03) at 16 weeks of gestation (Table 3). The aerobic group showed higher HDL difference values (−4.9 ± 5.6 vs. 7.2 ± 2.6; *p* = 0.03) relative to controls (Table 3). In intent-to-treat analysis, the aerobic group (*n* = 21), relative to controls (*n* = 11), exhibited a higher HDL change across gestation (*p* = 0.03). In the per protocol analysis at 36 weeks of gestation, exercisers have 21.2% lower maternal TG (245.3 ± 19.8 vs. 193.3 ± 14.0; *p* = 0.04) relative to controls (Table 3). In per protocol analysis, the control group exhibited a 106% increase in TG, while the aerobic group had a 92% increase (126.2 ± 14.4 vs. 89.8 ± 11.7, *p* = 0.06) across pregnancy (Table 3).

### 3.4. Correlations and Regression Analysis 

None of the potential covariates (maternal age, pre-pregnancy BMI, gravid, parity, VO2peak, GA, and GWG) impacted the regression coefficients; thus, they were excluded from the models. In an ITT analysis, there was a significant negative correlation between MET∙min∙week-1 and 36-week TG levels (rho_S_ = −0.368; *p* = 0.02); no other significant correlations were found between MET∙min∙week-1 and maternal lipids at 36 weeks of gestation. There were no significant correlations with measures of differences of DHA and EPA across gestation. After controlling for TG at 16 weeks’ gestation, there were no significant predictors of TG at 36 weeks’ gestation. After controlling for 36-week DHA and EPA levels, we found exercise dose was a negative predictor for 36 weeks’ TG levels (F (1,36) = 6.98; *p* = 0.01, β= −0.403, 95% CI= −0.253, −0.033), with an r^2^ of 0.16 (Table 4). After controlling for TG difference through pregnancy, we found exercise dose was a significant predictor for at 36 weeks’ TG levels (F (2,31) = 4.22; *p* = 0.02) with an r^2^ of 0.23. There were no other significant associations between MET∙min∙week-1 and maternal 36 weeks measures of TC, LDL, HDL, non-HDL, and TC/HDL ratio. There were no significant linear regression models of maternal DHA and EPA concentrations with MET∙min∙week-1 for maternal lipid measures of TC, LDL, HDL, TG, non-HDL, and TC/HDL ratio at 36 weeks of gestation (Table 4).

## 4. Discussion

The purpose of this study was to examine the influence of prenatal aerobic exercise and maternal concentrations of DHA and EPA on maternal lipid profiles in late pregnancy. We hypothesized that maternal aerobic exercise and higher DHA and EPA concentrations would be associated with improved, healthy maternal lipid profiles compared to non-exercising controls; and that prenatal exercise and DHA or EPA levels predict improved maternal lipid levels. The major findings of this study are: (1) maternal exercise dose is associated with and predicts lowered maternal TG levels at 36 weeks’ gestation; (2) maternal DHA and EPA concentrations did not exert measurable effects on maternal lipids at 36 weeks of gestation; and (3) prenatal exercise did not influence the relationship between maternal DHA and EPA concentrations and maternal lipids at 36 weeks of gestation.

Our finding that maternal exercise during pregnancy influenced maternal TG was expected. Consistent evidence shows exercise lowers serum TG concentrations in non-pregnant populations via enhanced skeletal muscle uptake of free fatty acids and increased whole-body post-exercise lipid oxidation [15,17,37,38,39,40]. Few studies previously examined the effects of chronic exercise on lipids in the pregnant populations [17,21,22,41]. For example, in a 12-week moderate exercise intervention, Ramirez et al. (2007) found lower TG levels in fasted samples among exercised-trained pregnant women tested between 28 and 32 weeks of gestation [21,41]. Our findings support those of Butler et al. (2004), which observed lower maternal TG concentrations in non-fasted samples during early pregnancy with increased self-reported moderate to vigorous physical activity [41]. Similar to the present study, Clark et al. (2019) implemented a RCT for 24 weeks with participants achieving 150 min of moderate intensity aerobic activity per week; however, they found higher TG levels at 36 weeks, most likely due to the samples being non-fasted [22]. Our results suggest prenatal exercise is beneficial for maternal TG during pregnancy and may explain improvements in pregnancy outcomes, such as delivering closer to full term (40 weeks). Maintaining healthy levels of maternal TG is clinically important because fetal fat deposition, which is heavily reliant on circulating levels of maternal TG, accelerates in late pregnancy. Although this study sampled mostly healthy pregnant women, these positive effects suggest that maternal exercise may provide pregnant women having abnormal levels of TG, as commonly found among those with obesity, with an effective strategy for normalizing TG levels, subsequently mitigating excess feta fat deposition. 

Contrary to the scientific literature, this study observed null associations between maternal levels of DHA and EPA and maternal lipid profiles in late pregnancy. Former studies showed negative associations between DHA/EPA and maternal TC, LDL, and TG, and a positive association with maternal HDL. Subsequent downstream effects include lower levels of adipocyte inflammation, oxidative stress, and free radical accumulation [25,26,28,29]. Previous studies reported consumption of DHA and EPA, either through supplementation or fish consumption, exerted positive effects on maternal lipids, reducing TC, LDL, and TG, and increasing HDL concentrations [23,24]. Despite the use of RBCs in measuring DHA and EPA in fasted blood samples, the null associations observed may be attributable to (1) a healthy sample, (2) DHA and EPA supplementation, and (3) lack of measurement of omega-6 and omega-3 fatty acids. The majority of our sample of pregnant women exhibited healthy pregnancies and an absence of existing chronic diseases prior to pregnancy. Additionally, these women received routine prenatal care providing them educational resources on the importance of proper nutrition, and on taking DHA and EPA to enrich prenatal vitamins during pregnancy. Consequently, the individual variability in maternal DHA, EPA, and lipid was reduced, which would negatively affect our statistical power in detecting statistically significant associations between DHA/EPA and maternal lipids. Another explanation for the observed null associations is our study did not measure maternal levels of omega-6 or omega-3 fatty acids. Omega-6 fatty acid concentrations are high in Western diets and often exceed the recommended ratio (1:1) with omega-3 fatty acids. A higher ratio of omega-6 to omega-3 fatty acids is shown to induce inflammation and adipogenesis. It is possible that higher levels of DHA and EPA are required to overcome the negative effects of higher ratios of omega-6 to omega-3 fatty acids. 

Uniquely, the current study evaluated the potential moderating effect of chronic, aerobic prenatal exercise on the association between maternal DHA/EPA and their impact on maternal lipids. Unexpectedly, chronic, aerobic prenatal exercise exerted no moderating effect on the relationship between maternal DHA/EPA and maternal lipids. We hypothesized that prenatal exercise would elicit a synergistic effect on this relationship, providing an optimal strategy for maintaining healthy levels of maternal lipids. Our small sample size, in addition to limited interindividual variability, likely resulted in the null observations. Another explanation for the null moderating effect of maternal exercise and the null association between DHA/EPA and maternal lipids is an insufficient exercise dose. Some studies suggest exercise doses exceeding ACOG guidelines of 500 MET∙min∙week^−1^ are required to elicit positive effects on maternal health outcomes, potentially for those who are metabolically challenged [22]; furthermore, a systematic review and meta-analysis support vigorous exercise as safe in the third trimester of pregnancy [42]. Thus, future studies should include samples that are larger and metabolically diverse, in addition to evaluating the impact of various exercise doses, not only on the primary outcomes of the current study, but in all studies evaluating the effects of prenatal exercise on maternal, fetal, and neonatal health outcomes. 

The current study possesses strength and weaknesses warranting attention. A strength of this study lies in its design as a prospective, randomized controlled trial, which is science’s most rigorous design and increases the strength of evidence towards causality. Second, quantifying DHA and EPA concentrations in RBCs, as opposed to plasma concentrations, provided longer-term concentrations of PUFAs in maternal circulation. Third, only fasted RBC samples were used, increasing the accuracy of maternal lipid profiles. Fourth, DHA and EPA concentrations were quantified using SPE and LC/MS/MS, a sensitive and precise technique of targeted metabolomics. Several potential limitations of this current study include: (1) the exclusion of 124 of participants in the early phase of this RCT due to non-fasted RBC blood samples; (2) a metabolically healthy sample of pregnant women; (3) the lack of a “true” control, which potentially diluted any effects of prenatal exercise, as the CON group participated in light-intensity stretching and breathing, with their leisure physical activity measured via self-reporting; and (4) the lack of measurement of other variables influencing maternal PUFA levels and/or lipid profiles. 

## 5. Conclusions

The current study strengthens the existing evidence for the positive effects of prenatal exercise on maternal TG in late pregnancy. Importantly, this observation suggests prenatal exercise may effectively normalize maternal lipids among pregnant women eliciting abnormal levels of TG (e.g., overweight or obese, gestational diabetes). Unexpectedly, our study showed no statistically significant association between maternal DHA/EPA and maternal lipid profiles, a contrary finding to the existing scientific literature. Moreover, our study explored the potential moderating effect of prenatal exercise on the association between maternal DHA/EPA concentrations and maternal lipids. However, no moderating effect was observed. Future studies should consider including samples that are larger, more metabolically diverse, and assess the effects of different exercise doses in order to maintain a healthy lipid profile, thus preventing negative pregnancy outcomes.

## Figures and Tables

**Table 1:** Intention-to-treat and per protocol analysis of maternal demographics between aerobic exercise and non-exercising controls.

		Intention-to-Treat			Per Protocol		
		Control (*n* = 11)	Exercise (*n* = 21)	*p*-Values	Control (*n* = 10)	Exercise (*n* = 20)	*p*-Values
Maternal Age (years)		31.2 ± 4.8	31.9 ± 4.7	0.71	30.5 ± 4.5	31.1 ± 4.5	0.72
Gravida (# of pregnancies) ^a^		2 (1.4)	2 (1.4)	0.52	2 (1.4)	2 (1.4)	0.24
Parity (# of births) ^a^		1 (0.2)	0.5 (0.2)	0.41	1 (0.2)	0 (0.2)	0.15
Pre Pregnancy BMI (kg/m^2^)		28.1 ± 4.8	25.6 ± 4.6	0.17	27.6 ± 4.7	25.4 ± 4.7	0.22
Pre-Intervention VO_2peak_ (mL/kg^−1.^min^−1^)		22.1 ± 3.6	23.6 ± 6.2	0.47	22.2 ± 3.8	25.3 ± 5.5	0.11
GWG (lbs.)		22.6 ± 8.8	32.7 ± 9.9	0.005 **	21.5 ± 8.6	31.7 ± 9.8	0.005 **
GA (weeks)		39.2 ± 0.7	39.7 ± 1.7	0.19	39.2 ± 0.8	40.1 ± 1.2	0.03 *

Values with normal distribution expressed as mean ± SD; ^a^ Not normally distributed-expressed as median (minimum, maximum); * *p* < 0.05; ** *p* < 0.01. BMI = body mass index, VO_2peak_ = peak volume of oxygen consumption, GWG = gestational weight gain, GA = gestational age at birth.

**Table 2:** Maternal DHA and EPA concentrations between aerobic exercise and non-exercising controls.

	Intention-to-Treat			Per Protocol		
	Control (*n* = 11)	Exercise (*n* = 21)	*p*-Values	Control (*n* = 10)	Exercise (*n* = 20)	*p*-Values
16 Week						
DHA (ng/mL)	2026.8 ± 1097.6	3702.0 ± 794.4	0.23	2024.3 ± 766.7	2673.8 ± 542.1	0.50
EPA (ng/mL)	938.9 ± 115.1	1114.1 ± 83.3	0.23	967.5 ± 99.9	1059.0 ± 70.7	0.34
36 Week						
DHA (ng/mL)	1812.3 ± 351.6	3056.0 ± 544.1	0.08	2230.4 ± 772.1	3143.6 ± 545.9	0.46
EPA (ng/mL)	977.3 ± 257.2	1148.4 ± 215.8	0.15	1335.1 ± 424.6	1369.3 ± 300.2	0.95
Difference						
DHA (ng/mL)	118.1 ± 412.9	−26.9 ± 540.8	0.98	206.1 ± 945.4	469.8 ± 668.5	0.82
EPA (ng/mL)	271.9 ± 241.5	102.3 ± 212.1	0.31	367.6 ± 422.2	310.3 ± 298.6	0.91

Values with normal distribution expressed as mean ± SD; DHA = docosahexaenoic acid, EPA = eicosapentaenoic acid.

**Table 3:** Maternal lipid profiles between aerobic exercise and non-exercising controls.

	Intention-to-Treat			Per Protocol		
	Control (*n* = 11)	Exercise (*n* = 21)	*p*-value	Control (*n* = 10)	Exercise (*n* = 20)	*p*-Value
16 Weeks						
TC (mg/dL)	211.8 ± 11.0	177.8 ± 7.9	0.01 *	198.2 ± 8.8	177.7 ± 7.1	0.08
HDL (mg/dL)	64.7 ± 3.4	64.4 ± 2.4	0.94	63.6 ± 3.8	64.0 ± 3.1	0.94
TG (mg/dL)	166.0 ± 29.2	102.8 ± 2.4	0.09	119.1 ± 12.3	97.2 ± 10.0	0.18
TC/HDL ratio	3.3 ± 0.2	2.8 ± 0.1	0.03 *	3.2 ± 0.2	2.8 ± 0.2	0.07
LDL (mg/dL)	111.0 ± 7.6	92.9 ± 9.3	0.06	111.0 ± 8.1	94.4 ± 6.7	0.13
Non-HDL (mg/dL)	140.0 ± 8.3	120.3 ± 6.2	0.07	131.2 ± 7.1	122.5 ± 6.0	0.36
36 Weeks						
TC (mg/dL)	241.0 ± 18.9	243.3 ± 8.7	0.90	252.4 ± 17.3	248.9 ± 12.3	0.87
HDL (mg/dL)	56.0 ± 21.6	62.6 ± 2.8	0.24	66.9 ± 4.7	65.8 ± 3.3	0.84
TG (mg/dL)	244.2 ± 30.8	212.3 ± 10.9	0.25	245.3 ± 19.8	193.3 ± 14.0	0.04 *
TC/HDL ratio	4.6 ± 0.4	4.1 ± 0.3	0.32	3.9 ± 0.4	4.0 ± 0.3	0.72
LDL (mg/dL)	131.4 ± 11.6	138.5 ± 8.5	0.62	136.4 ± 15.5	144.6 ± 11.0	0.67
Non-HDL (mg/dL)	175.1 ± 13.2	180.8 ± 9.6	0.73	175.1 ± 13.4	180.2 ± 11.2	0.77
Difference						
TC (mg/dL)	46.5 ± 14.7	69.3 ± 6.9	0.12	54.2 ± 14.4	65.2 ± 11.8	0.56
HDL (mg/dL)	−4.9 ± 5.6	7.2 ± 2.6	0.03 *	3.3 ± 3.4	5.3 ± 2.8	0.65
TG (mg/dL)	103.7 ± 15.6	112.7 ± 8.5	0.92	126.2 ± 14.4	89.8 ± 11.7	0.06
TC/HDL ratio	0.54 ± 0.4	0.59 ± 0.2	0.59	0.65 ± 0.2	0.79 ± 0.2	0.62
LDL (mg/dL)	19.3 ± 9.3	40.5 ± 7.0	0.08	25.4 ± 11.5	41.6 ± 9.4	0.29
Non-HDL (mg/dL)	41.6 ± 11.1	62.4 ± 8.1	0.14	41.6 ± 11.2	60.2 ± 9.4	0.21

Values with normal distribution expressed as mean ± SD; * *p*-value < 0.05. TC = total cholesterol, HDL = high-density lipoprotein, LDL = low-density lipoprotein, TG = triglycerides.

**Table 4:** Multiple linear regression models of maternal exercise dose, DHA concentration, and EPA concentration influence on maternal lipid profiles at 36 weeks of gestation.

		β (95% CI)	*p-*Value
Exercise Dose MET∙min∙week^−1^	36 Week TC (mg/dL)	0.035 (−0.064, 0.079)	0.84
	36 Week HDL (mg/dL)	−0.013 (−0.019, 0.018)	0.94
	36 Week LDL (mg/dL)	0.284 −0.008, 0.113)	0.09
	36 Week TG (mg/dL)	−0.403 (−0.253, −0.033)	0.01 *
	36 Week Non-HDL (mg/dL)	0.184 (−0.032, 0.108)	0.27
	36 Week TC/HDL ratio	0.196 (−0.001, 0.003)	0.25
36 Week DHA Concentration	36 Week TC (mg/dL)	−0.253 (−0.012, 0.002)	0.13
	36 Week HDL (mg/dL)	0.055 (−0.001, 0.002)	0.75
	36 Week LDL (mg/dL)	−0.270 (−0.010, 0.001)	0.11
	36 Week TG (mg/dL)	−0.081 (−0.014, −0.009)	0.74
	36 Week Non-HDL (mg/dL)	−0.247 (−0.011, 0.002)	0.14
	36 Week TC/HDL ratio	−0.197 (0.000, 0.000)	0.24
36 Week EPA Concentration	36 Week TC (mg/dL)	0.144 (−0.009, 0.023)	0.39
	36 Week HDL (mg/dL)	0.154 (−0.002, 0.006)	0.92
	36 Week LDL (mg/dL)	0.125 (−0.009, 0.018)	0.46
	36 Week TG (mg/dL)	−0.004 (−0.027, 0.027)	0.98
	36 Week Non-HDL (mg/dL)	0.100 (−0.011, 0.020)	0.56
	36 Week TC/HDL ratio	−0.036 (0.000, 0.000)	0.83

MET = metabolic equivalents of exercise, DHA = docosahexaenoic acid, EPA = eicosapentanoic acid, TC= total cholesterol, HDL = high-density lipoprotein, LDL = low-density lipoprotein, TG = triglycerides; * *p*-value < 0.05. Model = unadjusted main effects model.

## Data Availability

Not applicable.

## Abbreviations

AA	Arachidonic Acid
ACN	Acetonitrile
DHA	Docosahexaenoic Acid
EPA	Eicosapentanoic Acid
GA	Gestational Age
GLU	Glucose
GWG	Gestational Weight Gain
H_2_O	Water
HDL	High-Density Lipoprotein
IPA	Isopropanol
LC/MS/MS	Liquid Chromatography Tandem Mass Spectrometry
LDL	Low-Density Lipoprotein
MeOH	Methanol
PUFA	Poly-Unsaturated Fatty Acid
RBC	Red Blood Cell
RCT	Randomized Controlled Trial
RPE	Rating of Perceived Exertion
SPE	Solid Phase Extraction
TC	Total Cholesterol
TG	Triglycerides
